# Authoritarian Leadership in Organizational Change and Employees’ Active Reactions: Have-to and Willing-to Perspectives

**DOI:** 10.3389/fpsyg.2019.03076

**Published:** 2020-02-05

**Authors:** Jing Du, Nan Nan Li, Yuan Jing Luo

**Affiliations:** Economics and Management School, Wuhan University, Wuhan, China

**Keywords:** authoritarian leadership, job mobility, cognitive trust, employees’ active support, organizational change

## Abstract

Although prior studies have found the negative relation of authoritarian leadership with workplace outcome, authoritarian leadership styles are particularly prevalent in emerging markets. This study examines the effectiveness of authoritarian leadership in organizational change by considering two boundary conditions: low perceived job mobility among employees in have-to exchange situations and high cognitive trust in leaders in willing-to exchange situations. Based on a sample of 203 employees and their supervisors in 39 work teams in China, multilevel modeling identified a negative impact of authoritarian leadership on employees’ active support for organizational change. However, this negative effect disappeared when perceived job mobility was low and cognitive trust in the leader was high. The findings offer insights into the prevalence of authoritarian leadership in emerging markets despite negative impressions of this leadership style (Harms et al., 2018).

## Introduction

Studies have demonstrated that authoritarian leadership is negatively related to workplace outcomes such as team interaction, employees’ organizational commitment, task performance, helping, and vocalization behavior (Pellegrini and Scandura, 2008; Chan et al., 2013; Schuh et al., 2013; Chen et al., 2014; Duan et al., 2018; Harms et al., 2018; Shen et al., 2019). However, authoritarian leadership styles are still particularly prevalent in emerging markets (i.e., the Middle East, Pacific Asia, and Latin America; Harms et al., 2018). The factors that influence the effectiveness of authoritarian leadership should therefore be of great interest to organizational researchers. Chen et al. (2014) called for research on the conditions in which authoritarian leadership has less harmful or even beneficial influences on employee performance. They suggested that certain situational factors may explain the persistence of authoritarian leadership. Moreover, the effects of this style of leadership involve interactions with other potential factors, such as societal norms (e.g., one should work hard) and economic conditions (e.g., unemployment; Chen et al., 2014; Harms et al., 2018).

In response to this call, the present study explores the effectiveness of authoritarian leadership in organizational change considering two boundary conditions: perceived job mobility and cognitive trust in the leader. When employees under authoritarianism perceive low job mobility, they are more likely to *have to* actively participate in organizational change; by contrast, employees are more likely to be *willing to* follow their authoritarian supervisors to involve into organizational change when they trust in the leader.

Perceived job mobility reflects the favorability of the external job environment from the employees’ perspective (Wheeler et al., 2007). Compared to developed economies, labor markets are less structured and flexible in emerging markets (Peng et al., 2008). The lack of availability of alternative work is likely to force employees to stay with their leaders. Although studies have demonstrated the relationship of authoritarian leadership with employee negative perception (Chen et al., 2014), employees may actively support the organizational change unless they believe that alternative work opportunities exist. Therefore, the present study proposes that the effects of authoritarian leadership on subordinates’ active reactions are less negative in the presence of low perceived job mobility.

Trust in the leader refers to an individual’s trust in a specific supervisor, rather than general trust in colleagues and the organization as a whole (Luo, 2005). Studies of characteristics-based trust have revealed factors underlying perceived trustworthiness (Mishra, 1996), which include competence, ability, and expertise, that is, cognitive trust (Butler and Cantrell, 1984). Employees associate a leader high in expertise with an increased likelihood of the success of organizational change, which may in turn result in greater financial rewards. As monetary rewards have high valence for employees in emerging markets, cognitive trust based on a leader’s expertise and professional achievements may reduce the shadow of authoritarian leadership because of the potential link between professional achievements and monetary rewards (Du and Choi, 2010). A high level of cognitive trust by employees in their leaders cultivates perceptions that following these leaders will lead to better living conditions and prosperity. In short, cognitive trust in a leader can create a willing or receptive frame of mind among employees. Employees are more likely to be willing to engage with their supervisors in exchange for the solid payback derived from their supervisors’ expertise, even though the authoritarian style is not welcome (Blau, 1964; Liu et al., 2013).

This study makes two theoretical contributions. First, we draw from exchange theory to explain the interactive process through which an authoritarian leader is likely to have positively influence on their employees (Blau, 1964; Yoshikawa et al., 2019). Previous studies usually utilized intrinsic motivation theory to explore the effectiveness of authoritarian leadership (Harms et al., 2018). For example, authoritarian leaders injure followers’ intrinsic motivation by showing little respect for them, controlling work process, and lowering their contribution (Zhang et al., 2014). This study proposed that employees would likely to exchange with their authoritarian leaders by involving in organizational change to obtain job security and rewards.

Second, previous studies have focused on individuals’ voluntary exchange behaviors using exchange theory (e.g., Colquitt et al., 2013). This study explores both voluntary and compelled exchange simultaneously by identifying perceived job mobility and cognitive trust in the leader as two moderators that shape subordinates reaction to authoritarian leadership (Blau, 1964; Liu et al., 2013). Previous research have identified employee active support as the critical factor of the success of organizational change (Hornung and Rousseau, 2007; Furst and Cable, 2008). The present research tests the effects of authoritarian leadership on subordinates’ active change support. These effects take changed forms depending on two moderators, namely, perceived job mobility and cognitive trust in the leader. We empirically validate our theoretical propositions via multisource data collected from 203 employees of 39 work teams in China.

## Hypotheses

### Authoritarian Leadership and Employees’ Active Change Support

Authoritarian leadership is a leadership style that stresses personal dominance, strong centralized authority and control over subordinates, and unquestioning obedience (Cheng et al., 2004; Chen et al., 2014; Harms et al., 2018). Authoritarian leadership has been found to negatively influence outcome variables such as team interaction, organizational commitment, task performance, and extra-role performance (Chen et al., 2014). Consistent with these findings, the present study proposes a negative main effect between authoritarian leadership and employees’ active support for organizational change.

Organizational change produces technical, structural, and conceptual innovation. Such change requires employees to not only modify their work routines but also go beyond the call of duty (Herscovitch and Meyer, 2002; Farahnak et al., 2019). Given the inherent uncertainty of organizational change, active support from employees is critical for its success. However, employees under authoritarian leadership are less likely to perform additional behaviors because of the low level of reciprocity between authoritarian leaders and employees (Chen et al., 2014).

Reciprocity is one of the defining “rules” of exchange, especially functional exchange relationships (Blau, 1964; Emerson, 1976; Cropanzano and Mitchell, 2005). Reciprocity implies that a bidirectional transaction is required in an exchange: something must be given and received (Cropanzano and Mitchell, 2005). An inherent expectation of the social norm of reciprocity is that people will respond to each other in similar ways, such as responding to rewards and benevolence from others with similar effort, kindness, and loyalty or responding to harmful, hurtful acts from others with either indifference or some form of retaliation (Blau, 1964). An authoritarian leader behaves in a commanding and strongly controlling fashion, without expressing positive emotions or demonstrating amicable concern (Chen et al., 2014). Employees may perceive that their active and additional effort is unlikely to obtain payoff from the authoritarian leader (Blau, 1964; Yoshikawa et al., 2019), leading to the negative relationship between authoritarian leadership and employees’ active support for organizational change. Thus, the following hypothesis is proposed:

Hypothesis 1. Authoritarian leadership is negatively related to employees’ active support for organizational change.

### The Moderator of Perceived Job Mobility

Perceived job mobility is defined as an individual’s perception of available alternative job opportunities (Wheeler et al., 2007). It represents an employee’s assessment of the favorability and perceived ease of movement among organizations when scanning the external job environment: greater number of job alternatives and market opportunities leading to higher perceived job mobility (Hui et al., 1999). Previous studies have demonstrated that perceived job mobility weakens the relationship between job satisfaction and intent to stay in an organization (Trevor, 2001; Wheeler et al., 2007), as well as predicts less extra-role behaviors (Hui et al., 1999).

This study proposes that perceived job mobility is likely to moderate the negative relationship between authoritarian leadership and active support for change. During organizational change, fewer job alternatives would increase the opportunity cost of non-cooperation with the organizational change (Lee and Mitchell, 1994; Lee et al., 1999; Wheeler et al., 2007). Rejecting or neglecting to participate in organizational change may result in reduced pay raises, negative performance appraisals, and even unemployment (Wheeler et al., 2005).

When job alternatives are unavailable or undesirable, therefore, employees are likely to engage in exchange behaviors with the leader who can help them survive in an organization (Wheeler et al., 2005; Yoshikawa et al., 2019). Indeed, due to the unavailable outside job alternatives, current job position is even more valuable and precious. Based on the reciprocal norm of exchange (Blau, 1964), it is rational for employees low in job mobility to show support for critical events within the organization, such as organizational change. This implies that the original negative authoritarianism–employee reaction relationship is likely to be alleviated. Thus, the following hypothesis is proposed:

Hypothesis 2. Perceived job mobility moderates the negative relationship between authoritarian leadership and active support for organizational change such that the relationship is less negative when perceived job mobility is low than when it is high.

### The Moderator of Cognitive Trust in the Leader

Cognitive trust in the leader refers to trust grounded on performance-relevant cognitions, such as competence, expertise, responsibility, reliability, and dependability (McAllister, 1995; Schaubroeck et al., 2011). Employees’ beliefs about the leader’s ability or competence are the primary element of cognition-based trust in the leader (Schaubroeck et al., 2011). Tannenbaum et al. (1977) identified task competence as a more important factor in complying with an immediate supervisor’s request than the reward or the level of coercion.

This study proposes that cognitive trust in the leader is likely to diminish the negative relationship between authoritarian leadership and active support for change. Leaders with employees’ cognitive trust can initiate strong reciprocal leader–follower interactions (Colquitt et al., 2007). Any form of change brings both achievement and crisis. When employees have cognitive trust in their leaders, however, they are willing to be vulnerable to the leader’s actions because of the high confidence that the success and corresponding rewards are realizable (Ötken and Cenkci, 2012). For example, when the decision-making process is centralized coercion, employees believe their leaders have made sensible and correct decisions.

Authoritarian leaders with high levels of expertise may lead employees to success in organizational change, consequently satisfying the needs of subordinates. Because employees in emerging markets place great importance on monetary rewards, they are willing to participate in the exchange with their capable supervisors for purely economic reasons (Blau, 1964; Du and Choi, 2010; Yoshikawa et al., 2019). Therefore, employees with high levels of cognitive trust in their leaders’ expertise may be more likely to accept their supervisors’ authority and follow them. Thus, the final hypothesis is proposed:

Hypothesis 3. Employees’ cognitive trust in their leader moderates the negative relationship between authoritarian leadership and active support for organizational change such that the relationship is less negative when cognitive trust is high than when it is low.

## Materials and Methods

### Sample and Procedures

The present data were collected from supervisors enrolled on a training program in a Chinese university. Supervisors who had engaged in implementing organizational change (e.g., change in performance appraisal, process reengineering, and introduction of new tools or methods) were selected. With approval and support from the executives and employees, initial data were collected from 220 employees and their supervisors (90% response rate). To protect the confidentiality of responses, each respondent received an envelope to seal the completed questionnaire. Records with unsealed and broken-seal envelopes, unmatched supervisor–subordinate pairs, less than 1 year of company tenure, and groups with fewer than three members were eliminated (Du and Choi, 2010). This screening procedure resulted in a final analysis sample of 203 employees from 39 work teams. The size of the teams in the final sample ranged between 3 and 11 members, excluding team leaders, with a mean of 6 (*SD* = 2.27). This sample consisted of 46.8% males, with an average age of 29.94 years; 45.3% of the sample was unmarried. The average organizational tenure was 3.76 years. The education level of the participants was diverse and included middle school (1%), high-school graduate (16.2%), 2 years of college (26.6%), bachelor’s degree (50.7%), and master’s degree (5.4%).

### Measures

Authoritarian leadership, perceived job mobility, and cognitive trust in one’s leader were reported by employees, whereas employees’ active support for organizational change was evaluated by their direct supervisors. All items were assessed on five-point Likert-type scales (ranging from 1 = “strongly disagree” to 5 = “strongly agree”).

#### Authoritarian Leadership

Authoritarian leadership was measured using three items (α = 0.86) from the scale developed by Cheng et al. (2004). The items were as follows: (a) “My supervisor asks me to obey his/her instructions completely”; (b) “My supervisor makes all decisions in our team, whether they are important or not”; and (c) “My supervisor always has the last say in meetings.”

#### Perceived Job Mobility

A three-item measure (α = 0.70) of perceived job mobility was adopted from the turnover literature (Hui et al., 1999; Wheeler et al., 2007). The items were scored in reverse, including (a) “Right now, it’s necessary for me to stay with this organization”; (b) “It’s hard to find job alternatives better than the current one”; and (c) “It’s very inconvenient for me to switch to another company.”

#### Cognitive Trust in One’s Leader

Cognitive trust in the leader was measured using three items (α = 0.91) adapted from the scale developed by McAllister (1995). The items were as follows: (a) “My supervisor approaches his/her job with expertise, professionalism, and dedication”; (b) “My supervisor possesses strong work ability”; and (c) “My supervisor convinces me of his/her capability.”

#### Active Support for Organizational Change

Employees’ behavioral support for organizational change was measured using three items (α = 0.88) taken from Herscovitch and Meyer (2002). The items included (a) “This employee actively accepts organizational changes”; (b) “This employee actively accepts changes to rules and requirements”; and (c) “This employee actively participates in organizational changes.”

### Control Variables

To control for potential effects of demographic factors on employees’ active change behavior, age, gender, education, work experience, organizational tenure, and group size were included in the analysis. Age was measured in years; gender was coded 0 for female and 1 for male; tenure with the company was measured in years; and education was coded 1 for middle school, 2 for high school, 3 for 2-year college, 4 for bachelor’s degree, and 5 for master’s degree.

## Results

The empirical distinctiveness of the study variables, i.e., authoritarian leadership, perceived job mobility, and cognitive trust in the leader, was examined by confirmatory factor analysis (CFA). The CFA results are shown in Table 1. The three-factor model for the variables reported by employees produced a significantly better fit [χ^2^ (df = 19) = 54.98, *p* < 0.001; CFI = 0.96, RMSEA = 0.09] than the two-factor model [combining perceived job mobility and cognitive trust in the leader, χ^2^ (df = 21) = 129.78, *p* < 0.001; CFI = 0.88, RMSEA = 0.16] and the one-factor model [χ^2^ (df = 22) = 426.95, *p* < 0.001; CFI = 0.57, RMSEA = 0.30]. The means, standard deviations, and inter-scale correlations for all study variables are reported in Table 2.

**TABLE 1:** Means, standard deviations, and correlations of the variables (*N* = 203).

**Variables**	**Mean**	***SD***	**1**	**2**	**3**	**4**	**5**	**6**	**7**	**8**
1. Age	29.9	5.09	–							
2. Gender	0.47	0.50	0.06	–						
3. Tenure	3.76	4.48	0.42***	–0.07	–					
4. Education	4.32	1.09	–0.06	–0.13	0.08	–				
5. Authoritarian leadership	3.21	0.81	0.02	0.11	–0.04	–0.07	–			
6. Perceived job mobility	2.54	0.69	0.03	–0.07	–0.08	0.19**	–0.02	–		
7. Cognitive trust in leader	4.21	0.64	–0.09	0.00	–0.01	–0.13	−0.19**	−0.21**	–	
8. Active support for organizational change	3.95	0.53	–0.05	–0.05	0.11	–0.03	−0.14*	–0.01	0.11	–

*p < 0.05; **p < 0.01; ***p < 0.001.

**TABLE 2:** Hierarchical linear modeling results.

	**Behavioral support for organizational change**				
***Individual-level predictors***	**Null model**	**Model 1**	**Model 2**	**Model 3**	**Model 4**
Age		0.00	–0.00	0.00	0.00
Gender		–0.04	–0.04	–0.04	–0.04
Tenure		–0.01	–0.01	–0.01	–0.01
Education		0.02	0.03	0.03	0.03
Authoritarian leadership		−0.08**	−0.07*	−0.09*	−0.08*
Perceived job mobility (PJM)			0.07		0.07
Cognitive trust in leader (CTL)				–0.03	–0.01
Authoritarian leadership × PJM			−0.11**		−0.09*
Authoritarian leadership × CTL				0.12*	0.11*
Sigma squared	0.16	0.16	0.16	0.16	0.16
Tau	0.12	0.13	0.13	0.13	0.13
Pseudo *R*^2^		0.04	0.00	0.00	0.00

*p < 0.05; **p < 0.01.

Taking into account the nested structure of the current data, with 203 employees of 39 work teams, we conducted Chi square tests of between-group variance and the results showed that the percentage of total variance that resides between groups is significant for employees’ active support for organizational change [40%, χ^2^ (28) = 192.11, *p* < 0.001]. We further calculated authoritarian leadership’s within-group agreement (*r*_wg_ = 0.95), intra-class correlations [ICC(1) = 0.15 and ICC(2) = 0.61], and the *F*-statistics (*F* = 2.11, *p* < 0.001), demonstrating satisfied group-level sharedness and mean difference among groups, although we focused on employees’ perceived authoritarian leadership at individual level. Therefore, a multilevel analytic approach was employed [hierarchical linear modeling (HLM), Raudenbush and Bryk, 2002] that considered shared variance among employees from the same team as well as non-independence of employee ratings offered by the team leader. The group mean centering method was adopted for both independent variables and moderators (Du and Choi, 2010).

Hypothesis 1 proposed a negative effect of authoritarian leadership on employees’ active support for organizational change. As reported in Model 1 in Table 2, after controlling for company, age, gender, organizational tenure, and education, the effect of authoritarian leadership on employees’ behavioral support for organizational change was significant (β = −0.08, *p* < 0.01). Thus, Hypothesis 1 is supported.

**FIGURE 1:**
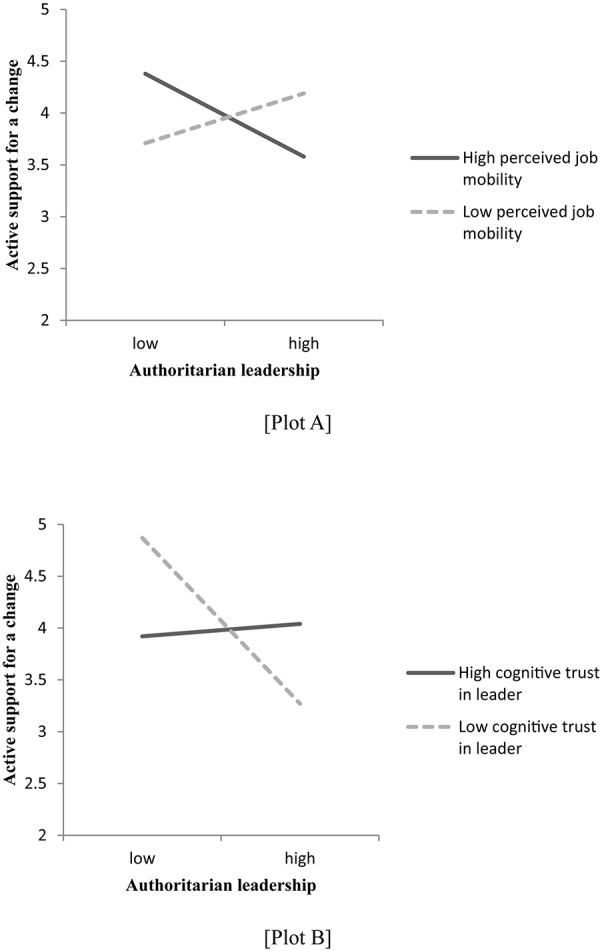
Moderation by perceived job mobility and cognitive trust in leader.

Hypothesis 2 proposed that perceived job mobility moderates the negative effect of authoritarian leadership on employees’ reactions. This hypothesis was tested in Model 2 in Table 2. The results showed that the individual-level interaction between perceived job mobility and authoritarian leadership was significantly related to employees’ active support for organizational change (β = 0.11, *p* < 0.01). The significant interaction was plotted by simple slope analysis (Aiken and West, 1991). Plot A in Figure 1 shows that the relationship between authoritarian leadership and active support for organizational change was negative when perceived job mobility was high (*b* = −0.20, *p* < 0.05) and neutral when perceived job mobility was low (*b* = 0.12, *ns*). This pattern confirms Hypothesis 2.

In Hypothesis 3, cognitive trust in one’s leader was proposed to alleviate the effect of authoritarian leadership on employees’ active support for organizational change. As Model 3 in Table 2 illustrates, the negative main relationship was moderated by employees’ cognitive trust in the leader (β = 0.12, *p* < 0.05). Plotting of this significant interaction (see Plot B in Figure 1) by simple slope analysis (Aiken and West, 1991) revealed that authoritarian leadership had a negative effect on employees’ active support for organizational change when employees’ cognitive trust in the leader was low (*b* = −0.40, *p* < 0.01) and a neutral effect when cognitive trust was high (*b* = 0.03, *ns*). These results demonstrate that employees with high cognitive trust in the leader showed less negative reactions to authoritarian leadership, confirming Hypothesis 3. The results of the model integrating authoritarian leadership, perceived job mobility, cognitive trust in the leader, and the interaction terms between these factors (see Model 4 in Table 2) supported all hypotheses.

## Discussion

Chen et al. (2014) issued a specific call to examine the situational influence on the effectiveness of authoritarian leadership. Following this call, this study sheds light on the relationship between authoritarian leadership and employees’ active reactions during organizational change considering two boundary conditions. This study involved the HLM analysis of 203 employees from 39 work teams in China. The results demonstrated that the negative relationship between authoritarian leadership and employees’ active support for organizational change was diminished when employees’ perceived job mobility was low and when their cognitive trust in the leader was high.

### Theoretical Implications

Based on intrinsic motivation theory, researchers have identified the negative influence of authoritarian leadership on employee outputs in the workplace (Zhang et al., 2014). However, practitioners in emerging markets continue to rely on authoritarian leadership with varying levels of success (Pellegrini and Scandura, 2008; Shen et al., 2019). Drawing from exchange theory (Blau, 1964), this study demonstrated that the positive relationship between authoritarian leadership and employee active support for organizational change support is possible. Followers would likely to follow their authoritarian leaders to obtain the valuable job security and financial rewards. Recent studies indeed found various influences of authoritarian leadership utilizing different theoretical explanations. Bodla et al. (2019) identified curvilinear relationships between authoritarian leadership and organizational citizenship behavior toward one’s supervisor using both intrinsic motivation theory and exchange theory. Wang and Guan (2018) proposed that authoritarian leader may enhance followers’ outputs by setting high-level goals. Using 211 supervisor–subordinate dyads data, they indeed found that authoritarian leadership is positively associated with employee performance and learning goal orientation mediates this relationship (Wang and Guan, 2018). Future research should shed light more on the effectiveness of authoritarian leadership using various theories.

Prior research has generally been leader-centered to explore how leaders affect employees’ perception of leadership behavior, such as affective trust in leader (Chen et al., 2014). The present study focused on both leader-centered and follower-centered perspectives. Regarding leader-centered perspective, this study proposes that cognitive trust to leaders is the general willing-to situation under which the negative authoritarian leadership effectiveness was diminished. The expertise of a supervisor can breed cognitive trust in the leader among subordinates, thus compensating for the shortcomings of authoritarianism by providing a promising future. This mechanism may not only apply to authoritarian leadership but may also act as a functional situational condition for other styles of leadership, such as abusive leadership (Tu et al., 2018). As a supplement to leadership style, expertise is rewarded with cognitive trust and hence strengthens the positive influence and ameliorates the negative influence of a leader’s characteristics and behaviors.

In terms of follower-centered perspective, the present study considers perceived job mobility as a have-to situation under which authoritarian leadership would like to positively influence followers. Exchange theory has generally been viewed voluntarily (e.g., Colquitt et al., 2013; Liu et al., 2013), and few studies have offered a have-to situation regarding the leadership effectiveness. The have-to situation indicates that leadership effectiveness is likely to be constrained by follower work environment as well, besides the favorable leader–member relationship. Thus, in addition to voluntary exchanges with supervisors, there are situations in which subordinates are compelled to show exchange behaviors (Trevor, 2001; Wheeler et al., 2007). Adopting perceived job mobility as an indicator of employees’ have-to exchange situation, the results demonstrated that employees who perceive few alternatives in the external work environment have no choice but to adapt to the *status quo* to continue exchanging with the leader. These findings reveal a new research field of non-spontaneous or non-voluntary exchange behaviors in have-to situations in relation to leadership effectiveness.

### Practical Implications

Power distance and leader benevolence may enhance the acceptance of authoritarian leadership in emerging markets (Farh et al., 2006; Chen et al., 2014; Harms et al., 2018). The present study proposes perceived job mobility as an additional explanation of the greater prevalence of authoritarian leadership in emerging markets. The low opportunity for movement in emerging markets strongly influences employees’ decisions and behavior. However, it is important to note that emerging markets are becoming more efficient, resulting in a narrowing range of applications of authoritarianism. Therefore, we see more leadership transferring from authoritarianism to transformational style.

Our findings suggest that expertise and work competence are critical for effective leaders. Scholars indeed have identified the three leadership skills including conceptual skills, technical skills, and human skills (Harrison et al., 2018). Interactive communication with followers about the knowledge in work domains, professional decision-making, and displaying working skills could develop employees’ cognitive trust to their leaders. Employees high in cognitive trust are more likely to follow their leaders because of the greater possibility of success and rewards.

### Limitations and Future Research

This study has several limitations that should be considered in interpreting its findings. First, the sample included only 203 employees from 39 work teams in China, which may limit the generalizability of the results to other cultural contexts (Harms et al., 2018). Replicating the present investigation in different cultures and work settings with larger samples and pursuing further validation of the present findings would be worthwhile.

Second, the present study utilized cross-sectional data and thus failed to support definite conclusions about causation or rule out the possibility of reverse causation. Employee displays of willingness or compliance may reinforce the representation of the authoritarianism of their leaders. Future research should use a longitudinal research design to evaluate the issue of causation.

Third, this study adopted three-item scales from previous researches to measure all variables. Although previous studies utilized the short-scale strategy to reduce the burden of responders and demonstrated satisfied validly (Choi, 2007), our approach still raised the critical issue of measurement validity. Both the independent variable and moderators were self-reported with a common-source bias. Full-item scales and multiple data resources should be employed to enhance the validity of measurement and reduce the common-source bias respectively in future study.

Despite these limitations, the present study provides new insights into the boundary conditions of authoritarian leadership effectiveness in organizational change by suggesting that low perceived job mobility places employees in have-to exchange situations, whereas high cognitive trust in the leader creates willing-to exchange situations. This research provides an intriguing starting point for researchers interested in the field of authoritarian leadership. First, to fully capture the boundary conditions of authoritarian effectiveness, future research should attempt to identify further characteristics that are relevant within the culture of emerging markets, which may moderate the effects of leadership behavior (Zhang et al., 2014). Such moderators may include employee self-complexity or individual values, such as the traditional Chinese “middle way” of thinking (Wheeler et al., 2005; Chen et al., 2014; Zhou et al., 2019).

Second, future research might investigate the potential multilevel dynamic interaction of authoritarian leadership with the emerging collective perception of employees. For example, employees’ individual trust may lead to the emergence of collective properties over the long term, and members of the same team may develop a greater level of homogeneity with respect to their cognitive trust in their leaders. Authoritarian leadership might influence work outcomes differently under multilevel situations or with different audiences.

## Data Availability Statement

The raw data supporting the conclusions of this article will be made available by the authors, without undue reservation, to any qualified researcher.

## Ethics Statement

The studies involving human participants were reviewed and approved by the Ethics Committee of Wuhan University. The participants provided their written informed consent to participate in this study.

## Author Contributions

All authors contributed conception and design of the study, collected the database, performed the statistical analysis, and wrote the first draft and sections of the manuscript. All authors contributed to manuscript revision, read and approved the submitted version.

## Conflict of Interest

The authors declare that the research was conducted in the absence of any commercial or financial relationships that could be construed as a potential conflict of interest.
